# Commentary: Return to the past with a view to the future

**DOI:** 10.1016/j.xjtc.2021.10.016

**Published:** 2021-10-16

**Authors:** Leonardo A. Mulinari, Tomas A. Salerno

**Affiliations:** a Section of Pediatric and Congenital Cardiac Surgery, Division of Cardiothoracic Surgery, University of Miami Miller School of Medicine and Jackson Memorial Hospital, Miami, Fla; b Section of Adult Cardiac Surgery, Division of Cardiothoracic Surgery, University of Miami Miller School of Medicine and Jackson Memorial Hospital, Miami, Fla

**Figure:**
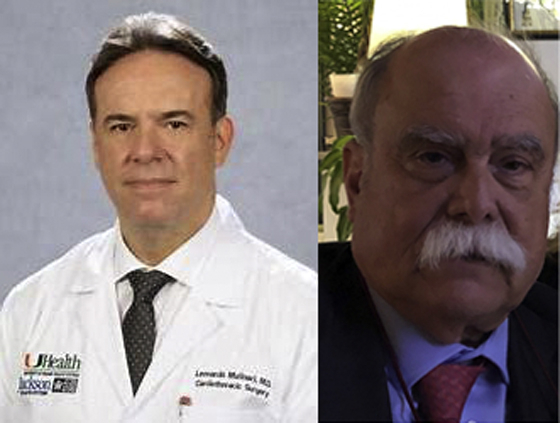
Leonardo A. Mulinari, MD, PhD, and Tomas A. Salerno, MD

**Central Message**

The history of mitral repair is reviewed and mitral folding plasty variations are described.

See Article page 68.

To perform an adequate mitral valve repair, a surgeon must not only be experienced but also he or she needs to know all concepts of mitral valve repair techniques. This allows the surgeon to use the perfect technique for each type of mitral valve pathology. Carpentier and colleagues^1^ developed a collection of maneuvers that allows for the achievement of mitral valve repair of most degenerative mitral diseases. Some procedures are simple and are more commonly used, such as quadrangular resection, and others are more complex. Among the initial mitral valve repair techniques, McGoon,^2^ more than 6 decades ago, utilized a folding technique for the prolapsed segment of the posterior mitral valve leaflet (folding plasty) as a method of repair the mitral valve.^2^ The simplicity of the procedure, reversibility of the initial folding plasty, if not perfect, and shortened operative times, made this a popular technique at that time. Since then, resectional techniques, with the introduction of neochordae, have greatly improved surgical repair techniques. For the most part, the McGoon folding plasty is of historical value, although there may be some situations in which it might be useful. Reasons for the difficulty in repair may be due to poor visibility/access using so-called minimally invasive techniques, and should not be a reason to compromise the repair in place of more advanced techniques. Tabata and colleagues^3^ describe in detail a variety of folding plasty techniques that the authors have utilized in the surgical treatment of posterior leaflet prolapse, reporting excellent results. Although this procedure was utilized for many years, long-term follow-up is lacking. Concern remains about bulky posterior leaflet after a folding plasty repair that may change the characteristics of the mitral valve, leading to problems in the long-term.

What is the value of this article? It is primarily a review of folding plasty techniques. To that end, it is excellent reading for those interested in the history of mitral repair. For surgeons with experience, in centers with a large volume of mitral surgery, it is likely that this article will not change their practice. However, in difficult cases, it remains an option, especially for surgeons who perform few valve repairs per year in their limited valve practice.
